# P-716. Epidemiology and patient burden of human parainfluenza virus in adults: a systematic review

**DOI:** 10.1093/ofid/ofae631.912

**Published:** 2025-01-29

**Authors:** Oliver Martyn, Peter Openshaw, Clemens Vlasich, Rolf Kramer

**Affiliations:** Sanofi Vaccines, Copenhagen, Hovedstaden, Denmark; Imperial College London, London, England, United Kingdom; Sanofi Vaccines, Copenhagen, Hovedstaden, Denmark; Sanofi Vaccines, Lyon, France, Lyon, Rhone-Alpes, France

## Abstract

**Background:**

The objective of this systematic review was to evaluate the scope of published epidemiological and patient outcomes data available for parainfluenza virus (PIV) in adults worldwide and to identify data gaps for this patient population.

**Methods:**

PubMed was searched for original articles on epidemiology or patient outcome of PIV (any strain) published from January 1, 2014, to August 26, 2023. This identified 549 publications. Studies were screened for relevant data in adults and in those at high risk for respiratory infections (severe pulmonary diseases, malignancies, transplants, cystic fibrosis, or those who were unhoused). Based on an initial screen, 390 articles were excluded, yielding 166 articles for full text review (**Figure**). This included 7 studies identified by screening bibliographies of excluded review articles that were not captured by the initial search. Upon review, 103 further articles were excluded (eg, study time frame was before 2014 or during the COVID-19 pandemic, lack of PIV or target data reported in adults). Collectively, this process identified a database of 63 articles for final analysis with any epidemiological or outcome data.

**Figure:**
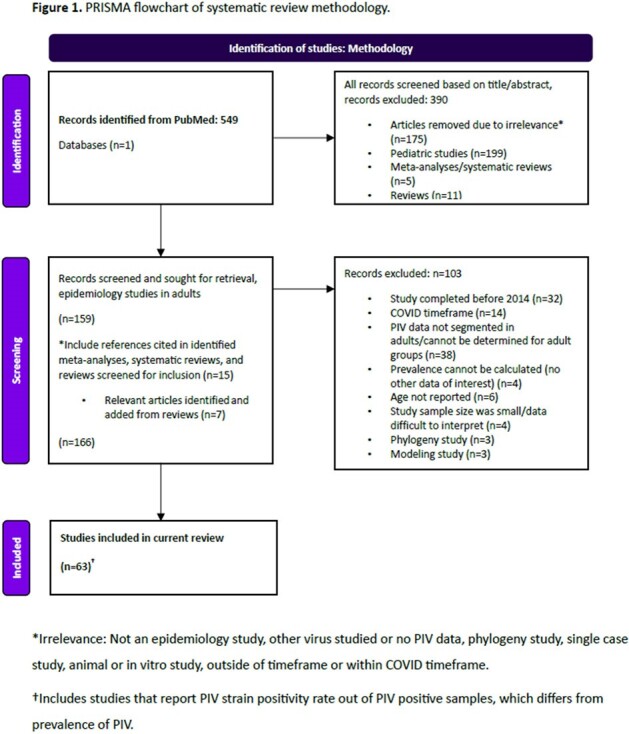

**Results:**

The prevalence of PIV reported in the literature varied widely based on geography and patient population. The population was broad including hospitalized adults, healthy volunteers, and those presenting with respiratory infections. Patients were defined as “otherwise healthy” if they were not considered high risk. Preliminary results indicate the overall prevalence of PIV (PIV1-4 or PIV unspecified) ranges from 0-15.2% [median 2%] in otherwise healthy adults, with PIV3 as the most prevalent (0.6-15.2% [2.9]) followed by PIV4 (0.4-5.9% [0.5]), PIV1 (0.5-2.8% [1.1]), and PIV2 (0-2.9% [1.1]). The prevalence of PIV was higher in at-risk adults (up to 52%). Patient outcome data was scarce, with mortality rates being the most frequently reported topic. Hematopoietic stem cell transplant patients were found to be the most at risk for PIV-associated mortality, though there was no clear strain-specific pattern of mortality given the limited data on patient outcomes.

**Conclusion:**

Significant data gaps remain on the burden of PIV on adults, particularly in otherwise healthy adults.

**Disclosures:**

**Oliver Martyn, MPH**, Sanofi: Employee of Sanofi|Sanofi: Stocks/Bonds (Public Company) **Peter Openshaw, PhD**, AstraZeneca: Advisor/Consultant|AstraZeneca: Honoraria|AstraZeneca: Speaker bureau participation|CSL Seqirus: Advisor/Consultant|CSL Seqirus: Honoraria|GSK: Advisor/Consultant|GSK: Honoraria|Icosavax: Advisor/Consultant|Janssen: Advisor/Consultant|Janssen: Honoraria|Medical Research Council UK: Grant/Research Support|Medscape: Speaker bureau participation|Moderna: Advisor/Consultant|Moderna: Honoraria|Pfizer: Advisor/Consultant|Pfizer: Honoraria|Sanofi: Advisor/Consultant|Sanofi: Grant/Research Support|Sanofi: Speaker bureau participation **Clemens Vlasich, MD, MSc**, Sanofi: Employee of Sanofi|Sanofi: Stocks/Bonds (Public Company) **Rolf Kramer, PhD**, Sanofi: Employee of Sanofi|Sanofi: Stocks/Bonds (Public Company)

